# Supplementary figures and images for: Docking studies and molecular dynamics simulations of the binding characteristics of waldiomycin and its methyl ester analog to Staphylococcus aureus histidine kinase
Source: PLoS One. 2020 Jun 5;15(6):e0234215. doi: 10.1371/journal.pone.0234215 (PMC7274439; doi:10.1371/journal.pone.0234215)

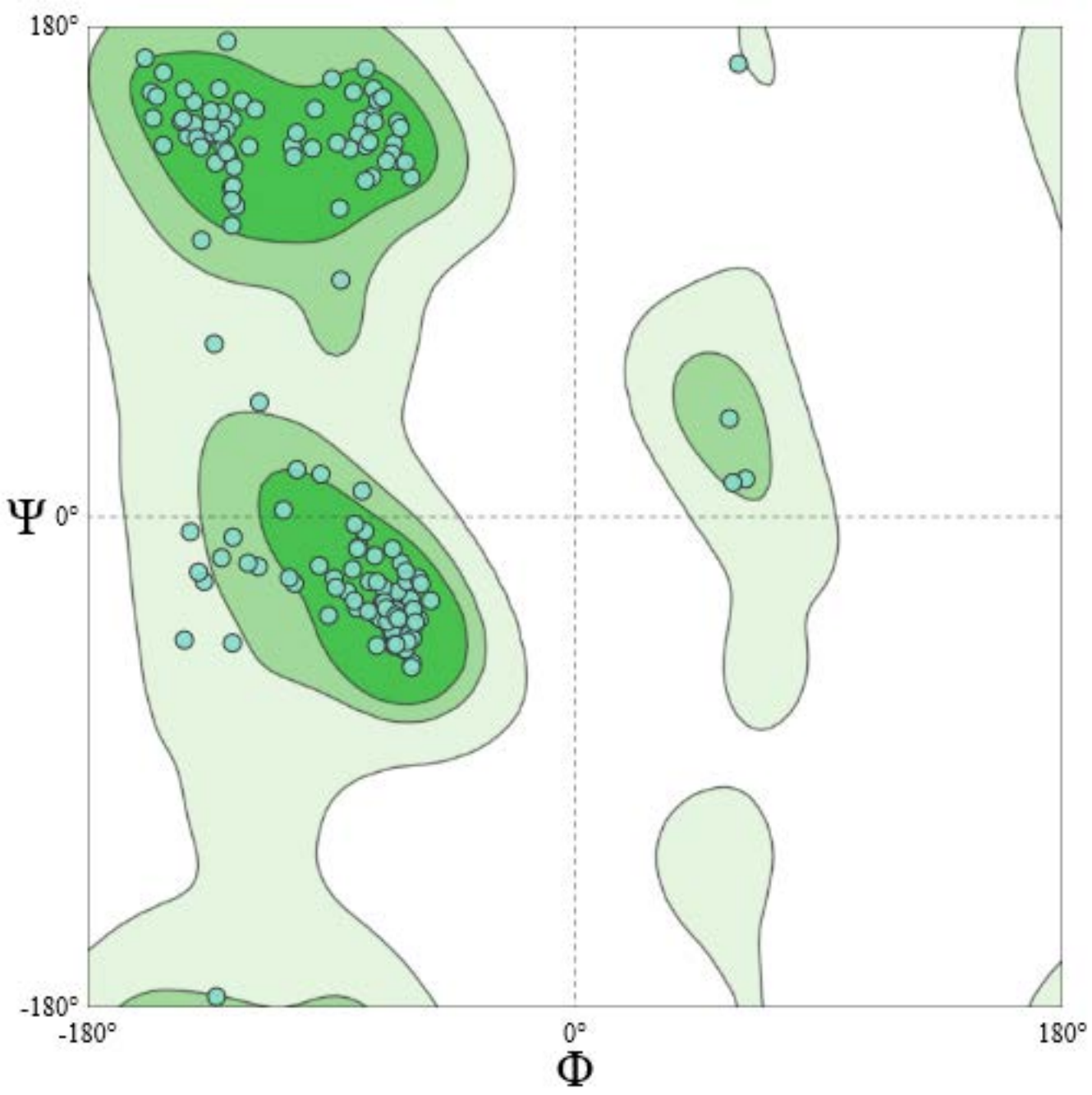

Supplement: S1 Fig — (PDF) [file pone.0234215.s001.pdf]

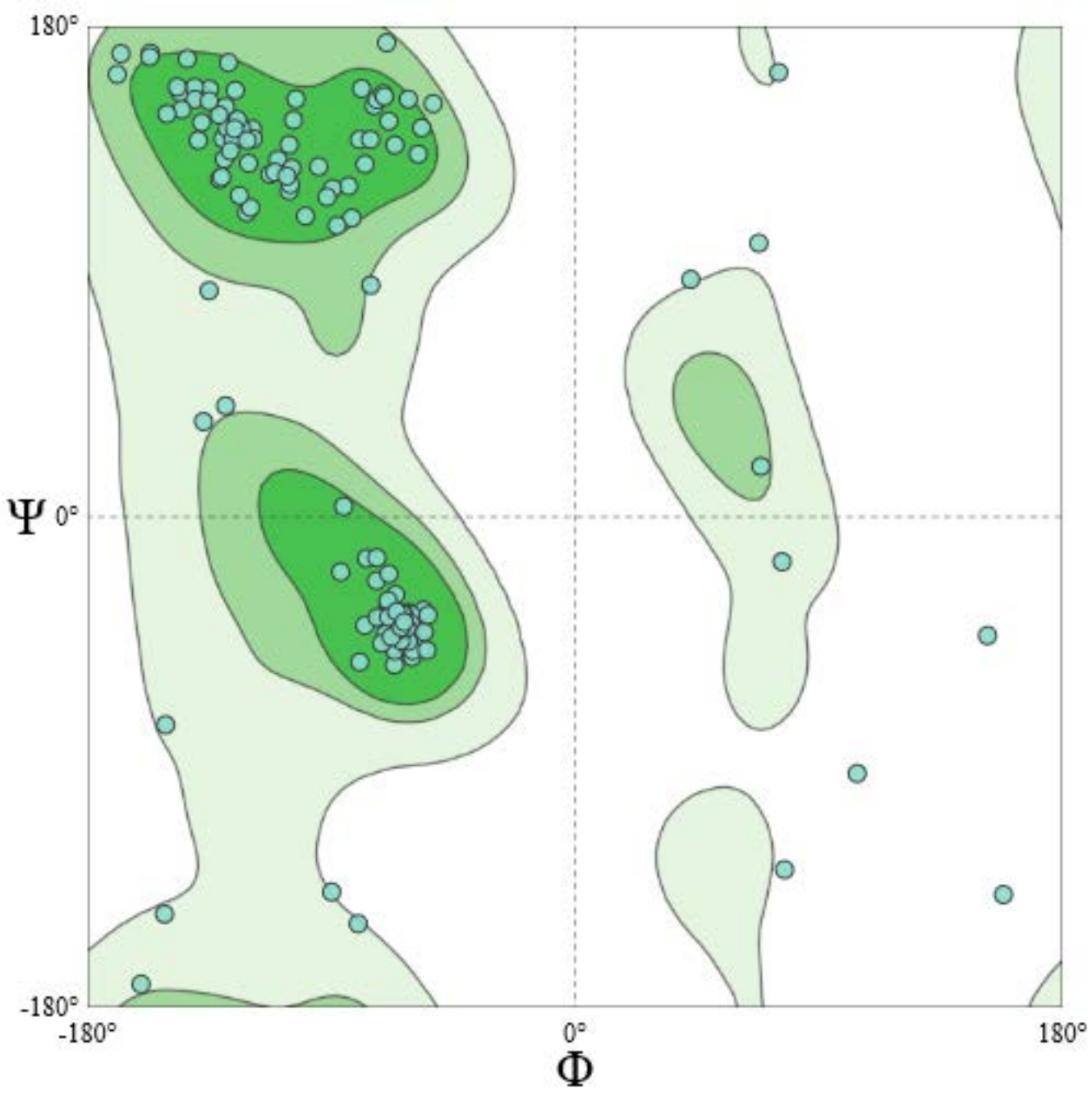

Supplement: S2 Fig — (PDF) [file pone.0234215.s002.pdf]

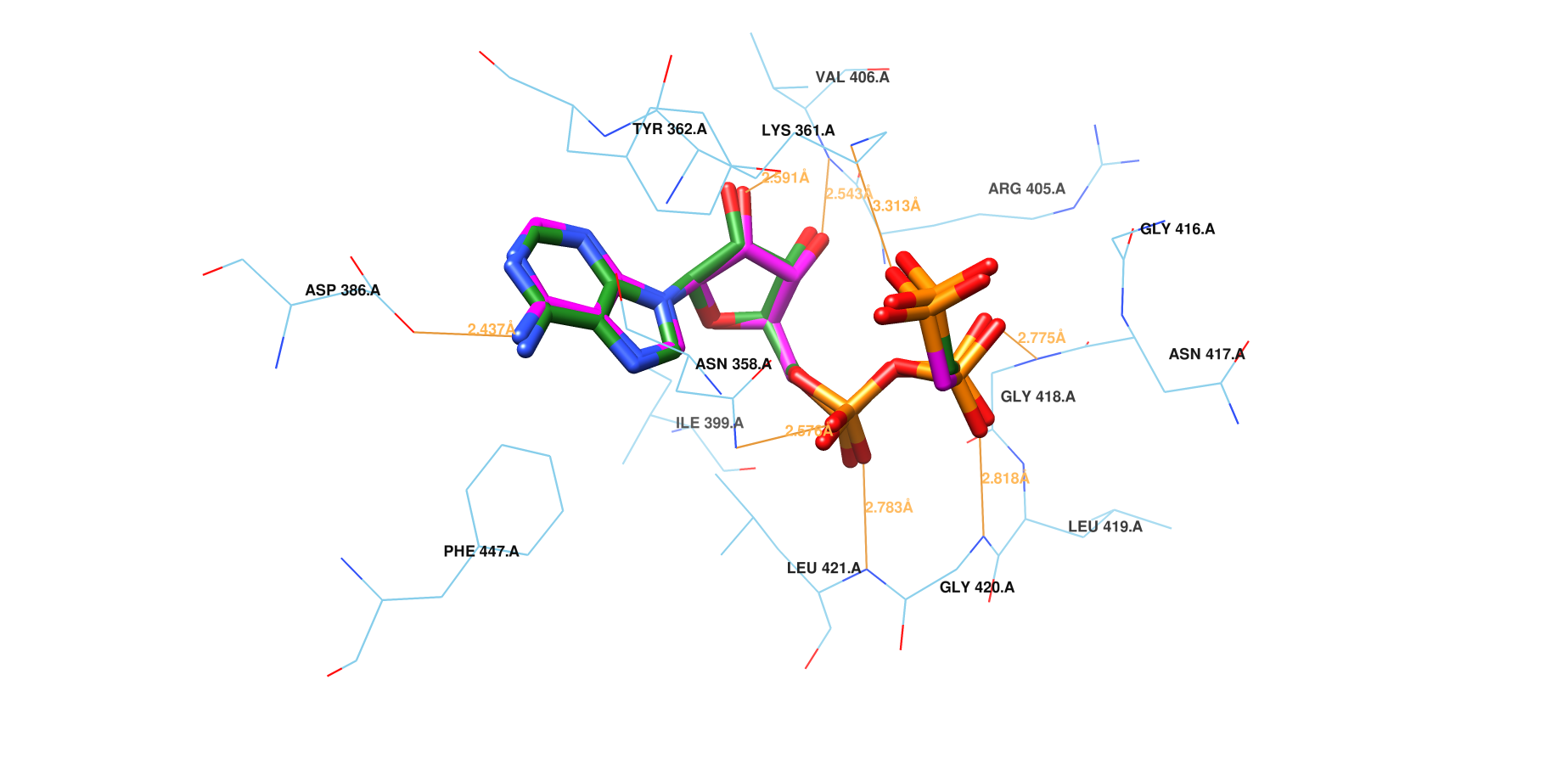

Supplement: S3 Fig — The co-crystallized ACP (from 5c93.pdb, colored magenta) and the redocked ACP structure (colored forest green), superimposed inside the binding site of 5c93. (TIF) [file pone.0234215.s003.tif]

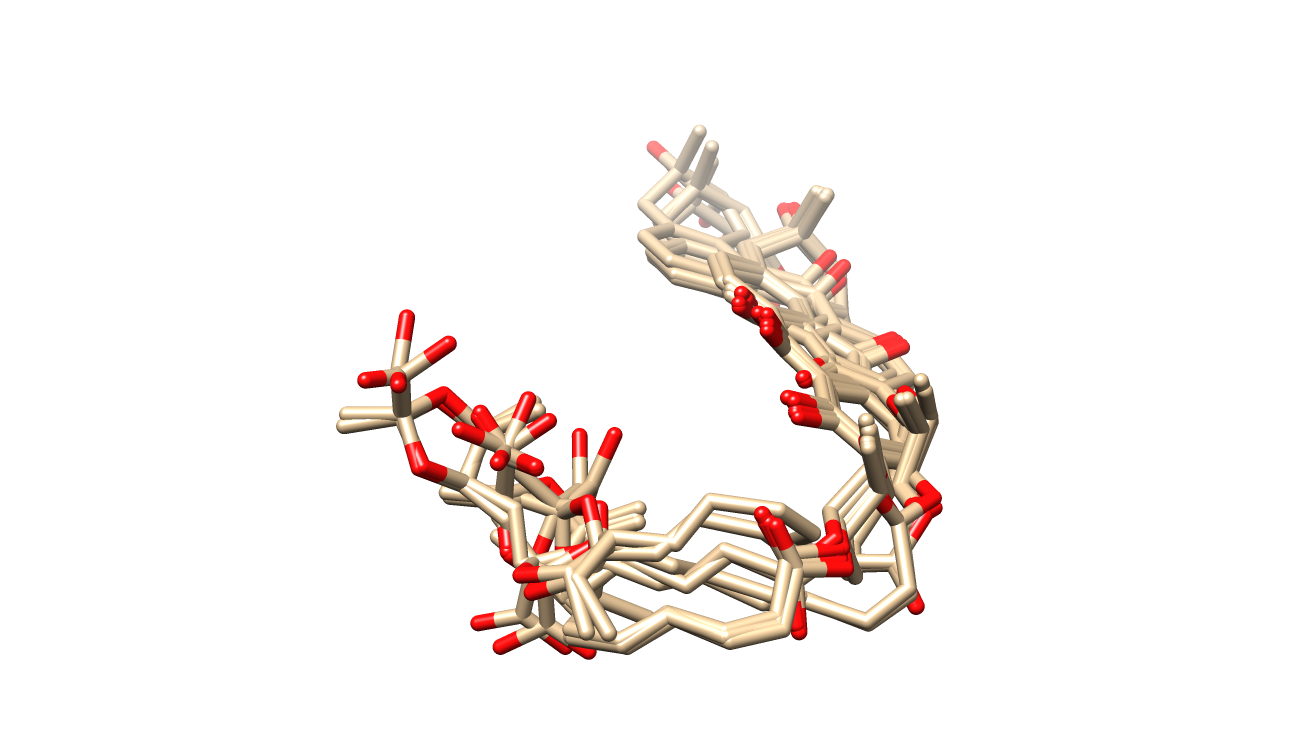

Supplement: S4 Fig — (TIF) [file pone.0234215.s004.tif]

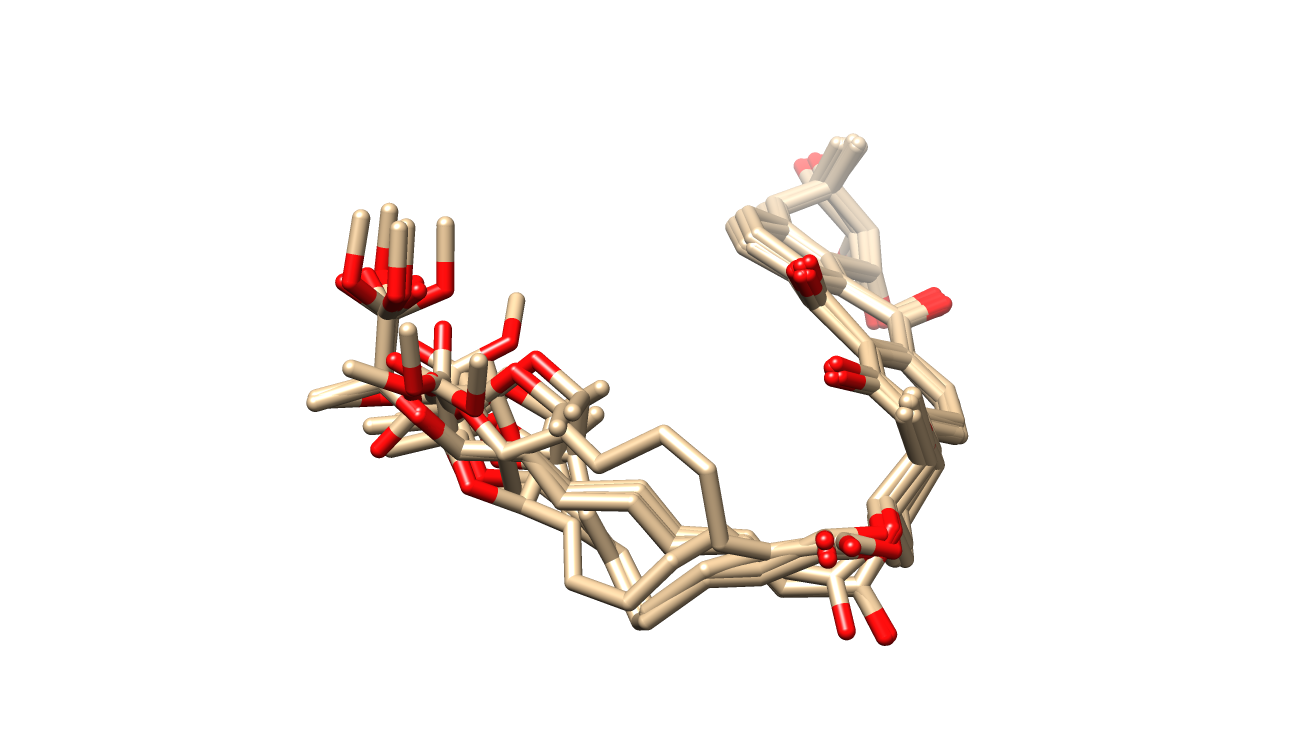

Supplement: S5 Fig — (TIF) [file pone.0234215.s005.tif]
