# Supplementary material for: Docking studies and molecular dynamics simulations of the binding characteristics of waldiomycin and its methyl ester analog to Staphylococcus aureus histidine kinase
Source: PLoS One. 2020 Jun 5;15(6):e0234215. doi: 10.1371/journal.pone.0234215 (PMC7274439; doi:10.1371/journal.pone.0234215)
Supplement: Attachment — Submitted filename: Response to Reviewers.docx [file pone.0234215.s008.docx]

Please include the following items when submitting your revised manuscript:

- A rebuttal letter that responds to each point raised by the academic editor and reviewer(s). This letter should be uploaded as separate file and labeled 'Response to Reviewers'.
- A marked-up copy of your manuscript that highlights changes made to the original version. This file should be uploaded as separate file and labeled 'Revised Manuscript with Track Changes'.
- An unmarked version of your revised paper without tracked changes. This file should be uploaded as separate file and labeled 'Manuscript'.

additional requirements.

1. Please ensure that your manuscript meets PLOS ONE's style requirements, including those for file naming. The PLOS ONE style templates can be found at https://journals.plos.org/plosone/s/file?id=wjVg/PLOSOne_formatting_sample_main_body.pdf and https://journals.plos.org/plosone/s/file?id=ba62/PLOSOne_formatting_sample_title_authors_affiliations.pdf

2.  We suggest you thoroughly copyedit your manuscript for language usage, spelling, and grammar. If you do not know anyone who can help you do this, you may wish to consider employing a professional scientific editing service.  

Whilst you may use any professional scientific editing service of your choice, PLOS has partnered with both American Journal Experts (AJE) and Editage to provide discounted services to PLOS authors. Both organizations have experience helping authors meet PLOS guidelines and can provide language editing, translation, manuscript formatting, and figure formatting to ensure your manuscript meets our submission guidelines. To take advantage of our partnership with AJE, visit the AJE website ([http://learn.aje.com/plos/](about:blank)) for a 15% discount off AJE services. To take advantage of our partnership with Editage, visit the Editage website (www.editage.com) and enter referral code PLOSEDIT for a 15% discount off Editage services.  If the PLOS editorial team finds any language issues in text that either AJE or Editage has edited, the service provider will re-edit the text for free.

Upon resubmission, please provide the following:

- The name of the colleague or the details of the professional service that edited your manuscript
- A copy of your manuscript showing your changes by either highlighting them or using track changes (uploaded as a *supporting information* file)
- A clean copy of the edited manuscript (uploaded as the new *manuscript* file)

Additional Editor Comments (if provided):


[Note: HTML markup is below. Please do not edit.]

Reviewers' comments:

Reviewer's Responses to Questions

**Comments to the Author**

1. Is the manuscript technically sound, and do the data support the conclusions?

The manuscript must describe a technically sound piece of scientific research with data that supports the conclusions. Experiments must have been conducted rigorously, with appropriate controls, replication, and sample sizes. The conclusions must be drawn appropriately based on the data presented.

Reviewer #1: Yes

Reviewer #2: Partly

2. Has the statistical analysis been performed appropriately and rigorously?

Reviewer #1: Yes

Reviewer #2: Yes

3. Have the authors made all data underlying the findings in their manuscript fully available?

The [PLOS Data policy](http://www.plosone.org/static/policies.action#sharing) requires authors to make all data underlying the findings described in their manuscript fully available without restriction, with rare exception (please refer to the Data Availability Statement in the manuscript PDF file). The data should be provided as part of the manuscript or its supporting information, or deposited to a public repository. For example, in addition to summary statistics, the data points behind means, medians and variance measures should be available. If there are restrictions on publicly sharing data—e.g. participant privacy or use of data from a third party—those must be specified.

Reviewer #1: Yes

Reviewer #2: Yes

4. Is the manuscript presented in an intelligible fashion and written in standard English?

PLOS ONE does not copyedit accepted manuscripts, so the language in submitted articles must be clear, correct, and unambiguous. Any typographical or grammatical errors should be corrected at revision, so please note any specific errors here.

Reviewer #1: Yes

Reviewer #2: Yes

5. Review Comments to the Author

Please use the space provided to explain your answers to the questions above. You may also include additional comments for the author, including concerns about dual publication, research ethics, or publication ethics. (Please upload your review as an attachment if it exceeds 20,000 characters)

Reviewer #1: The authors of this paper study the binding properties of waldiomycin and its methyl ester analog to histidine kinase (HK) of Staphylococcus aureus. To this purpose, the 3D structure of the catalytic domain of S. aureus HK was first generated using a homology model. Then, they study the docking of the two molecules and perform MD simulations on the more stable predicted complexes. Finally, the binding free energies of the two derivatives were calculated using the generalized Born surface area approach (MM-GBSA). According to the calculations, the higher affinity displayed by waldiomycin relative to its methylated analog can be explained because the carboxylate group of the former is engaged in a hydrogen bond with Lys100 of the protein. This stabilizing interaction is not possible in the methyl ester variant.
The work is well conducted, and the reviewer believes that it can deserve publication in PLOS ONE.
However, before it is published, authors should clarify the followings:
**Comment 1)** The homology model is based on a protein that presents only an identity of 36%. This result could lead to erroneous conclusions. This point should be explicitly discussed in the manuscript.

**Answer**;

Page 11 line: 231 >> The sentence, mentioned below, was added including references and subsequent references were consecutively renumbered.

“Generally, it is assumed that two proteins topologies are usually similar once their sequences share about 30% identity [35]. Moreover, Dimitrii et al developed structures of average quality (RMSD < 4.0 Å) based on target-template sequence identity ≥ 20% [36].”

**Comment 2)** Is there any experimental evidence that supports the significance of Lys100 for the recognition of waldiomycin?

**Answer**; Through the literature survey, I couldn’t find any experimental evidence that supports the significance of Lys100 for the recognition of waldiomycin.

**Comment 3)** The MM-GBSA approach does not consider the entropy associated with the binding. This point should be discussed in the paper.

**Answer;** Page 16; Line 352 >> The following sentence was added with the corresponding reference and subsequent references were renumbered all over the manuscript.

“However, MM/GBSA approach does not consider the entropy associated with the binding, it is an efficient powerful tool for correct ranking of ligands. Although the inclusion of the conformational entropy is crucial for calculating absolute free energies of binding, it is not necessary for ranking the binding affinities of similar inhibitors [42].”

**Minor points:**
**Point 1)** Page 6. Lines 142-144. The following paragraph should be rewritten: 'Using the TIP3P water solvent model, the protein molecule was included in a water box with 10 Å thickness and using sodium ions were as counter ions and using explicit solvation'.

**Answer;** Page 6. Lines 141-143>> The paragraph was replaced with the paragraph mentioned below.

“The protein molecule was explicitly solvated with water molecules and sodium counter ions were also added to get neutral system. A water box with 10 Å thickness was created using the TIP3P water solvent model to completely surround the protein molecule."

**Point 2)** Page 7. Lines 163-164. The sentence is not correct. The GAFF force field is applied only for the ligands and not for the complexes. In this regard, what is the approach used to calculate the partial charges of the ligands (BCC, HF/6-31G*, etc.)? What is the starting structure used for these ligands? The X-ray structure of waldiomycin has been previously reported (deposition number: CCDC 892997)

**Answer**; Page 7. Line 164>> The word “complexes” is replaced with “ligands”.

<<>> BCC in antechamber module was used to calculate the partial charges of these ligands.

<<>> The starting structures are prepared in pdb format using Chemoffice package and mol2 files are prepared using UCSF-Chimera program.

<<>> “I didn’t use the x-ray structure of waldiomycin”

**Point 3)** The structure of the best ten candidates derived from the docking calculations, together with their corresponding score, should be included in the Supporting Information.

**Answer;** Page 13 Line 278>> The paragraph mentioned below was typed to fulfill the reviewer comment.

“The top scored 10 conformations of waldiomycin and waldiomycin ester are shown in S3 and S4 mol2 files respectively. The docking score and hydrogen bond information of these top scored 10 conformations are also listed in S5 and S6 txt files respectively.”

**Point 4)** The MD simulations were done for a short time (35 ns). What is the reason for using this simulation time?

**Answer;** Both the waldiomycin and the protein structures were equilibrated with unobvious RMSD fluctuations through the 35 ns time frames. Waldiomcyin ester structure was equilibrated after 14^th^ ns and still stable for more 20 ns. The md simulation stopped when both the system had reached their stable state with unobvious fluctuations.

**Point 5)** Page 12. The interaction between the ligands and the receptor should be explained in detail (hydrogen bonding, CH/p, etc.).

**Answer;** Page 13 Line 272-284>> The paragraph mentioned below was added

“The two binders were efficiently docked inside the binding site of HK/Walk structure. The binding poses of the two binders showed similar V-shaped orientations inside a binding site delineated with the side chains of residues Lys63, Lys100, Leu102, Hie103, Pro104, Leu131, Ile132, Asp135, Ile138, Lys139, Arg183, Val184, Asp185, Asn195. The hydrophobic part of the binders fit inside one binding pocket near Val184 while the carboxyl and ester groups of the two compounds, fit inside a second binding pocket near Lys100 (Fig 6a,b).

The top scored 10 conformations of the waldiomycin and waldiomycin ester are shown in S3 and S4 mol2 files respectively. The docking score and hydrogen bond information of these top scored 10 conformations are also listed in S5 and S6 txt files respectively. The Grid score of these binding poses is in the range value of -63.629486 to -62.780089 for waldiomycin and -66.089516 to -64.42131 for waldiomycin methyl ester which is un-comparable with the difference in their experimental inhibition of *S. aureus* HK/Walk, IC50 values 10.2 and 75.8 µM respectively. The top scored binding poses of the two binders were selected for further investigations using MD simulation.”

<<>> Page 15 Line 335-342>> Cpptraj hydrogen bond analysis are listed in Table 1 and shows the hydrogen bonds with high occupancy for the two simulation systems. The table shows two different hydrogen bond sets for the two complex systems. For waldiomycin-HK/Walk complex, the amino acid residues Lys63, Ser98, Lys100, Gln128, Asp135, Lys139 and Val184 showed hydrogen bond with the ligand. For waldiomycin ester-HK/Walk complex, the amino acid residues, Lys63, Leu102 and His103 showed hydrogen bond with the ligand. The hydrogen bond pattern difference between the two simulation systems is an indications of the two different binding conformations of waldiomycin and waldiomcyin ester analogue (Fig 8).

**Point 6)** Figure 2: the font used is tiny and difficult to read. Why are the authors showing two different binding sites?

**Answer;**

<<>> The fonts in Fig 2 were corrected to be more readable.

<<>> In the sketch representation of Fig 2, the use of different binding sites in the figure to designate the fact that during MD simulation there are three possibilities for the docked ligand. The first one, the ligand don’t change the initial docking site, second one, the ligand repositions itself into different binding site and third one the ligand exit outside its initial docking site.

**Point 7)** Figure 3: the double bond in one of the aromatic rings of both molecules is not adequately represented.

**Answer;** <<>> The double bond of the aromatic rings of both molecules was corrected in Figure 3

**Point 8)** Figure 5: the number are difficult to read. In Figure 5a, is it true that the red line corresponds to all atoms of the protein? There is a typo in the Figure caption: 'toms' should be 'atoms’.

**Answer;** <<Page 12 Line 259>>The typo in Figure 5a was corrected “red lines are for backbone and black lines for all atoms’ in the Figure 5a and Figure catption ‘toms’ was changed to ‘atoms’

**Point 9)** Figure 6: The labels are difficult to read.

**Answer;** <<>> The labels in the figure are corrected to be more readable. Also, In view the comment of Reviewer #2, Figure 6 was moved to the supplementary files and renamed as S7 and the subsequent figures are renumbered.

**Point 10)** Figure 7: An interaction diagram should be added for both complexes.

**Answer;**

<<>> Page 14 Line 277>> The figure 7 was renumbered as Fig 6

<<>>	Two diagrams for the two complexes were performed.

**Point 11)** Combine Figure 8 and 9 and use the same color code as the one used in Figure 9 to show the RMSD of the methylated derivative.

**Answer;** <<>> Figure 8 and 9 combined into one Figure and renumbered as Fig 7 using same color code to show the RMSD of the methylated derivative.

**Reviewer #2:** Bacterial histidine kinases (HK) are interesting targets for the design of antibacterial agents. The authors here report the computational studies of the binding to the S. aureus HK/Walk domain of two known inhibitors: waldiomycin and its methyl ester analogue. The interest of the research is based in the difference in the potency of these two ligands: waldiomcyin presents 7.4‐ fold stronger potency for over waldiomycin ester. The authors provide a 3D perspective about their biding modes correlated with this affinity difference. They propose a homology model of the S. aureus HK enzyme and use it for docking and MD simulations. The computational work has been conducted in a professional manner. The here reported studies are relevant in the context of finding novel antimicrobial agents and providing new insights into the SAR of waldiomycin as S. aureus HK inhibitor.

However, the results are not sufficiently well detailed and discussed and some additional studies are missed.

**Comment**: The antimicrobial activity (MIC) of waldiomycin is reported in ref. 20 (Igarashi et al., 2013). At the conclusions, the authors state that “waldiomcyin presents 7.4‐ fold stronger potency for over waldiomycin ester”. This statement should be justified in the main text and also the context where “potency” is used. In any case, the concepts of “affinity” and “potency” are mixed along the manuscript. This should be revised and clarified.

**Answer**;

- Page 2 line 40. Page 5 Line 107. Page 5 Line 116. Page 13 Line 294. Page 17 Line 368. Page 18 Line 384. One term “potency” is used.

**Comment**: Line 87. “Unfortunately, few inhibitors of TCS have been described against S. aureus, a major pathogen with high virulence factor”. A reference should be cited here. Which inhibitors are these? They should be docked in order to compare with waldiomycin. For example, the compounds cited in ref. 20 (Igarashi et al., 2013) could be good examples to be docked and compared with waldiomycin.
**Answer**;

- The compounds cited in ref 20 (Igarashi et al 2013) include the two compounds used in our study however other compounds tested in this paper were completely inactive.

**Comment**: Also the lack or low binding of waldiomycin towards human kinases could be studied (docking) as control, in order to avoid secondary/toxic effects. Lys100 was found to be crucial residue for S aureus HK binding of waldiomcin. Is this Lys present in human kinases?

**Answer**;

- Page 3 Line 60, 61. Bacteria respond to the extracellular changes through **TCS pathways, which are absent in humans** and are mediated by histidine kinases (HKs) [5].

**Comment**: The discussion about the docking poses and the results about the binding pose after the MD simulation should be enriched: more details about the interactions, pockets and residues that are important for the binding. It is not clearly mentioned which is the protonation state considered for waldiomycin (protonation state under physiological condition). Is it docked as carboxylate or as carboxylic acid? The authors mention the H-bond with Lys100 (from the figures, it seems that with the side chain ammonium). This interaction drives the carboxylic/carboxylate group towards the Lys100 ammonium, while in the case of the waldiomycin methyl ester is not clear which interactions are not stable, and which new interactions are stablished in the new binding pose after the simulation (why now the new interactions do stabilize the new pose).

**Answer**;

- Waldiomycine was used, as input to tleap program in Amber tools, with protonation state under physiological PH. However, it showed electrostatic bond between its Oxygen atom and the Nitrogen atom of the amine group of the side chain of Lys10; that wasn’t happened with the waldiomcyin ester group.
- In view of the comment of Reviewer #1, the top scored 10 conformations of waldiomycin and its methyl ester was submitted as supporting mol2 files S3 and S4 respectively. Also, S5 and S6 txt files were submitted as supporting information files including Docking scores and Hydrogen bond between the poses and protein atoms.
- The new interactions with waldiomycin ester are declared in (Method section: Page 9 Line 195 – 201. Results and discussions section: Page 15 Line 335 – Page 16 Line 350.)
- *I suppose that the electrostatic binding with Lys100 from one end of the molecule and h-bond with Val184 from the other end of the molecule, anchor the molecule in its V shaped conformation inside the binding site, also with the aid of other h-bonds. On the other hand, the absence of this interaction between Lys100 and waldiomycin ester released one end of the structure to free its strain from the V-shaped into the extended shape conformation*.

**Comment**: Why does waldiomycin methyl ester migrates to other binding pocket? Was this pocket reached by any predicted docked pose? This compound has a reported low affinity but it remains bound during the simulation. What does the energetics analysis say about the interactions? The contributions to the binding free energy from each residue should be analyzed from the MM-GBSA and discussed in the light of the new binding pose for the waldiomycin methyl ester.

**Answer**;

- The electrostatic interaction developed between waldiomycin carboxylic oxygen atom and the nitrogen atom of side chain amino group of Lys100 is stronger than the hydrogen bond type.
- Waldiomycin ester didn’t form such bond which is a potential reason for it leave out of the binding site after short time of md simulation (cf waldiomycin).

**Comment**: Can the authors deepen into the required interactions for a ligand to be a good *S. aureus* HK inhibitors?
There is ongoing work to use the obtained information for the design of potential HK/Walk inhibitors.

**Answer**;

- In view of the valuable comment of the Reviewer, we added the studies of a) Hydrogen bond analysis using cpptraj module in amber; b) Pairwise per-residue interaction energy decomposition using mmpbsa module in ambmer:
- Method section: Page 9 Line 195 – 201.
- Results and discussions section: Page 15 Line 335 – Page 16 Line 350.
- The obtained information is used in an ongoing work to discover new lead compounds through the virtual screening of the databases and we are still looking for partner laboratory to make in vitro screening of the developed molecules.

**Minor corrections**

-Line 279-284. Refs 37 and 38 referring to limitations in ligand-protein docking (2012 and 2002) and ref 39 as publication to illustrate advances in MD simulations (2011) are obsolete. More recent references should be cited.

**Comment**: -FIG5.
(a) Scale of the time should be adapted at the actual time of the simulation (0 to 30 ns?).

**Answer**;

- Fig 5a is a result of MD simulation for 10 ns “used for optimization of the homolog model” (35 ns MD simulations were performed for complexes Fig 8 only).

**Comment**: (b) The residue numbers should (x-axis) should be cut before 250. A label should be added indicating this corresponds to residue numbering.

**Answer**;

- Corrections were typed to Fig 5b

**Comment**: -FIG.6 should go to SI. It´s a calculation made as validation, it´s not a main result of the work.
**Answer**;

- Fig 6 was moved to SI and took number S7, and the subsequent figures were renumbered in sequence.

**Comment**: -Typos:
Line 90- “the 3D structure is unavailable in protein databank”.
It probably should say “the 3D structure is unavailable in the protein databank”. The PDB web page should be added as reference.

Answer;

- Page 4 Line 90. Correction and web page address was added.

Additional work is required, which I think is necessary for the sake of sound conclusions, impact of the work and usefulness to the scientific community. In view of the comments noted above, I would recommend its acceptance for publication in PLOS ONE only when the corrections have been addressed.

6. PLOS authors have the option to publish the peer review history of their article ([what does this mean?](https://journals.plos.org/plosone/s/editorial-and-peer-review-process#loc-peer-review-history)). If published, this will include your full peer review and any attached files.


If you choose “no”, your identity will remain anonymous but your review may still be made public.


**Do you want your identity to be public for this peer review?** For information about this choice, including consent withdrawal, please see our [Privacy Policy](https://www.plos.org/privacy-policy).

Reviewer #1: No

Reviewer #2: No


[NOTE: If reviewer comments were submitted as an attachment file, they will be attached to this email and accessible via the submission site. Please log into your account, locate the manuscript record, and check for the action link "View Attachments". If this link does not appear, there are no attachment files to be viewed.]


While revising your submission, please upload your figure files to the Preflight Analysis and Conversion Engine (PACE) digital diagnostic tool, <https://pacev2.apexcovantage.com/>. PACE helps ensure that figures meet PLOS requirements. To use PACE, you must first register as a user. Registration is free. Then, login and navigate to the UPLOAD tab, where you will find detailed instructions on how to use the tool. If you encounter any issues or have any questions when using PACE, please email us at [figures@plos.org](mailto:figures@plos.org). Please note that Supporting Information files do not need this step.
