# Supplementary material for: Docking studies and molecular dynamics simulations of the binding characteristics of waldiomycin and its methyl ester analog to Staphylococcus aureus histidine kinase
Source: PLoS One. 2020 Jun 5;15(6):e0234215. doi: 10.1371/journal.pone.0234215 (PMC7274439; doi:10.1371/journal.pone.0234215)
Supplement: Attachment — Submitted filename: Response to Reviewers.docx [file pone.0234215.s009.docx]

**Author’s responses to the reviewers’ comments**

Reviewers' comments:

Reviewer's Responses to Questions

**Comments to the Author**

1. If the authors have adequately addressed your comments raised in a previous round of review and you feel that this manuscript is now acceptable for publication, you may indicate that here to bypass the “Comments to the Author” section, enter your conflict of interest statement in the “Confidential to Editor” section, and submit your "Accept" recommendation.

Reviewer #1: All comments have been addressed

Reviewer #2: All comments have been addressed

2. Is the manuscript technically sound, and do the data support the conclusions?
The manuscript must describe a technically sound piece of scientific research with data that supports the conclusions. Experiments must have been conducted rigorously, with appropriate controls, replication, and sample sizes. The conclusions must be drawn appropriately based on the data presented.
Reviewer #1: Yes
Reviewer #2: Yes

3. Has the statistical analysis been performed appropriately and rigorously?
Reviewer #1: Yes
Reviewer #2: Yes

4. Have the authors made all data underlying the findings in their manuscript fully available?
The [PLOS Data policy](http://www.plosone.org/static/policies.action#sharing) requires authors to make all data underlying the findings described in their manuscript fully available without restriction, with rare exception (please refer to the Data Availability Statement in the manuscript PDF file). The data should be provided as part of the manuscript or its supporting information, or deposited to a public repository. For example, in addition to summary statistics, the data points behind means, medians and variance measures should be available. If there are restrictions on publicly sharing data—e.g. participant privacy or use of data from a third party—those must be specified.
Reviewer #1: Yes
Reviewer #2: Yes

5. Is the manuscript presented in an intelligible fashion and written in standard English?
PLOS ONE does not copyedit accepted manuscripts, so the language in submitted articles must be clear, correct, and unambiguous. Any typographical or grammatical errors should be corrected at revision, so please note any specific errors here.
Reviewer #1: Yes
Reviewer #2: Yes

6. Review Comments to the Author
Please use the space provided to explain your answers to the questions above. You may also include additional comments for the author, including concerns about dual publication, research ethics, or publication ethics. (Please upload your review as an attachment if it exceeds 20,000 characters)

Reviewer #1: All my comments have been addressed, and the manuscript is now acceptable for publication.
Minor points:
**1) Units of the grid score should be indicated in both the main text and Supporting Information.

***Answer:***

>>Page 14, line 284,285: The unit, “kcal mol^–1^ “, was included in the main test; and also in S6 and S7 files of the Supporting Information.

**2) Units of pairwise energy decomposition should be shown in Table 1 and the main text. Is this energy given in Kcal or Kcal/mol? (see main text: 'range value -2.722 to -4.371 Kcal (Table 1)'

***Answer:***

>>Page 17, line 348. The unit was changed into “kcal mol^-1^).

Reviewer #2: The authors have successfully addressed all the concerns and corrections.
Minor correction:
Line 301: Units should be specified. And probably E values can be rounded to two decimals (this is sufficiently precise in this order of magnitude).

***Answer:***

>>Page 18, lines 362, 363. E values were rounded to two decimals and units “kcal mo^l-1^” were specified.

7. PLOS authors have the option to publish the peer review history of their article ([what does this mean?](https://journals.plos.org/plosone/s/editorial-and-peer-review-process#loc-peer-review-history)). If published, this will include your full peer review and any attached files.
If you choose “no”, your identity will remain anonymous but your review may still be made public.
**Do you want your identity to be public for this peer review?** For information about this choice, including consent withdrawal, please see our [Privacy Policy](https://www.plos.org/privacy-policy).
Reviewer #1: No
Reviewer #2: No
